# Microprojectile plant transformation for sugarcane giant borer pest management

**DOI:** 10.1186/1753-6561-8-S4-P101

**Published:** 2014-10-01

**Authors:** Felipe Redorat, Fernando Fonseca, Leonardo Macedo, Isabela Lourenço, Maria Fátima Grossi-de-Sá

**Affiliations:** 1 EMBRAPA - Cenargen, Universidade de Brasilia (UnB), Brasilia,Brazil; 2 EMBRAPA - Cenargen, Universidade Católica de Brasilia (UCB), Brasilia, Brazil

## Background

The sugarcane giant borer, *Telchin licus licus*, is the major insect pest of crops in northern and northeastern regions of Brazil. This insect has a long life cycle, which lasts about 160-190 days and presenting four major life stages, egg, larvae, pupae and moth [1]. During larval stage, the insect penetrates the plant as soon as they hatch and starts feeding of the stalk, where it stays for a period of 100 to 120 days, approximately. Moreover, it allows the penetration of opportunistic organisms such as fungi and bacteria which ferment the sugarcane juiceand prevents its use in industrial processes. There are no known commercial plants resistant to this insect and the use of chemical pesticides has been inefficient due it´sendophytic behavior. The use of biotechnological tools such as RNA interference and expression of proteins with insecticidal activity has allowed developing alternative methodologies for pest control [2]. One of those alternatives is the transformation of plants aimingthe expression of molecules that targets insect survival genes, causing problems in its development and increasing the mortality rate. Proteins with insecticidal activity, as Cry proteins have been used by researches for a long time to increase resistance against agricultural pests [3], since after ingestion of transgenic plants expressing such molecules, the toxin causes an osmotic lysis of midgut cells leading to insect death. The present work focuses on the transformation of elite events of sugarcane to improve plant resistance to the giant borer *T. licus licus*.

## Methods

Sugarcane variety RB855156 was used for this work, as it represents one of the most cultivated sugarcane plants in Brazil. Meristematic cells were collected and subsequently grown in tissue culture to induce embryogenic callus formation [4]. Two constructions for dsRNA expression in sugarcane plants targeting specific *T. licus licus *developmental genes, and one construction that induces the production of a Cry toxin with high activity against the insect larvae were used. All constructions presents ammonium gluphosinate herbicide resistance gene. M10 tungsten particles were shot against60 embryogenic callus per plate. A total of eight plates were shot with dsRNA I construction, nine containing dsRNA II and four containing a Cry toxin gene. After DNA bombardment the material was placed in regeneration medium with ammonium gluphosinate1.5 mg/L. After three months 118 regenerated seedlings were sprayed with the herbicide Finale^® ^(1%).

## Results and conclusions

After the selection process, five dsRNA I, two dsRNA II and eight Cry plants survived. All plants confirmed to be PCR positive for the presence of the transgenes, presenting an average transformation efficiency of 0.02% plants/callus. This study was the first to be conducted in order to improve sugarcane resistant against *T. licus licus*. Southern blot experiments are being carried out to confirm the introduction of the expression cassette into the plants genome. The best events will be tested in bioassays to determine resistance levels against sugarcane giant borer larvae.
